# Diving Deep: Exploring Dual Palatal Canals in the Upper Second Molar

**DOI:** 10.7759/cureus.61266

**Published:** 2024-05-28

**Authors:** Khyati Manik, Anuja Ikhar, Aditya Patel, Manoj Chandak, Jay Bhopatkar, Priyanka R Bhojwani, Pratik Rathod

**Affiliations:** 1 Department of Conservative Dentistry and Endodontics, Sharad Pawar Dental College and Hospital, Datta Meghe Institute of Higher Education and Research, Wardha, IND

**Keywords:** root canal treatment, magnification, endodontics, maxillary second molar, extra palatal canal

## Abstract

Dental anatomy exhibits considerable variation with the presence of additional canals being a common occurrence. The upper second molar typically presents with three canals and three roots; however, variations such as the existence of an extra canal or a root can pose challenges during endodontic treatment. Maxillary molar is characterized by an additional canal located within the palatal root, often exhibiting complex configurations and variations in morphology. Access refinement is critical to gaining adequate visibility and facilitating instrumentation. Meticulous exploration of the pulp chamber floor and careful examination of radiographs from different angles are essential for accurate diagnosis. Careful negotiation and cleaning of the extra canal with appropriate files and irrigants are essential to remove pulp tissue and debris effectively. Furthermore, obturation of the canal space with biocompatible materials is crucial to ensure a three-dimensional seal and prevent bacterial ingress. Clinically, the inability to detect and treat the extra palatal canal can lead to persistent infection, incomplete debridement, and compromised treatment outcomes. This case report delves into the significance of this anatomical variation, diagnostic modalities, and effective management strategies.

## Introduction

The successful outcome of endodontic treatment relies heavily on accurate diagnosis and thorough management of root canal anatomy. Among the most challenging variations encountered is the presence of an extra palatal canal in the maxillary molars, particularly the second molars. The importance of detecting extra canals in any tooth cannot be overstated, as it is critical for the success of endodontic treatment. Undetected extra canals can lead to incomplete cleaning and shaping, resulting in persistent infection and ultimately, treatment failure. Different anatomical pathways can aid in the detection of extra canals. One such pathway is the use of dentinal maps, which provide a detailed layout of the internal tooth structure. These maps can help identify additional canals that may not be visible through conventional radiographs. The presence of an isthmus, a narrow band of tissue connecting two or more canals, is another critical feature to examine. Isthmuses can harbor bacteria and debris, making their identification and cleaning vital for successful treatment.

The intricate structural variations within the canals of the root present a persistent problem for endodontic practitioners. While the maxillary second molar typically exhibits a three-root, three-canal configuration, deviations from this norm are not uncommon. One such variation is the existence of an extra canal within the palatal root, known as the palatal two canal. Despite its rarity, the identification and thorough management of this anatomical anomaly are critical for the success of endodontic therapy in the maxillary second molar. Despite the overall low prevalence of anatomical variations in the palatal canal of maxillary molars (less than 2%), certain populations exhibit higher rates. For instance, the prevalence is 6% in the maxillary second molars of the Chinese population [1] and 14% in the maxillary second molars of the Indian population [2]. Managing an extra palatal canal in a maxillary molar requires a methodical approach to ensure comprehensive treatment. Firstly, accurate diagnosis is paramount, utilizing advanced imaging techniques such as cone-beam computed tomography (CBCT) alongside meticulous examination of preoperative radiographs.

Most research on anatomical variation in molars seems to focus on the first molar. There are not as many studies on anatomical variations in other molars. Fava et al. reported a single root and a single canal in the upper second molar of a similar patient in which anatomical variation was also present in the upper first molar [3], while Alani came across a total of four roots in a similar patient in the upper second molar of both the upper quadrants [4]. Christi et al. stated a total of 14 cases with two palatal roots of the upper second molar over 40 years [5]. In 2012, Shojaeian et al. described a case of two palatal canals in the upper second molar tooth [6].

Magnification plays a pivotal role in enhancing the clinician's ability to detect and navigate intricate root canal anatomy, particularly in challenging cases like the presence of an extra palatal canal. In the case of maxillary molars, where palatal canals can exhibit complex morphology, magnification aids in visualizing fine details and distinguishing between main canals and accessory canals. Magnification aids in minimizing procedural errors by providing a clear and magnified view of the root canal system. This reduces the likelihood of missed canals, ledge formation, perforations, and other iatrogenic complications associated with endodontic treatment. An upper second molar with two separate palatal canals is described in this case report.

## Case presentation

**Figure 1:**
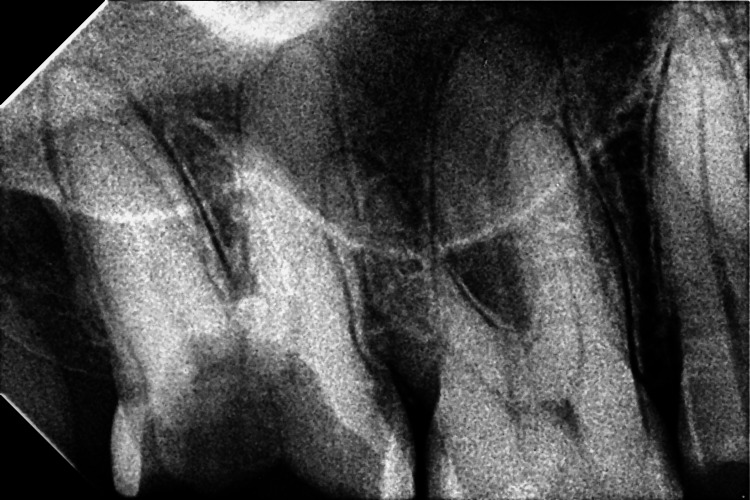
Shows preoperative radiograph with the upper right second molar

A 38-year-old female patient, living in Wardha, contacted the Department of Conservative Dentistry and Endodontics of Sharad Pawar Dental College and Hospital complaining of pain in the right upper back of the jaw for a month. When the chief complaint was specified, the pain was spontaneous or persisted for minutes after removing the stimulus (usually heat, less often cold). The past medical as well as dental history was non-significant. While examining clinically, deep occlusal caries was seen with the upper right second molar with tenderness on percussion positive. Radiolucency involving enamel, dentin, and pulp, as well as periodontal ligament space widening with the right upper second molar, was detected on X-ray examination. Four distinct roots, each with its canal, were observed on the radiograph, corresponding to Vertucci's type I classification. A pulpal neurosensibility test was performed. On the electric pulp test, the upper right second molar shows a delayed response in comparison to the adjacent and contralateral tooth. A diagnosis of symptomatic irreversible pulpitis with apical periodontitis was made for the upper right second molar (Figure 1). A treatment plan was formulated and explained to the patient, and the procedure commenced following the patient's informed consent.

**Figure 2:**
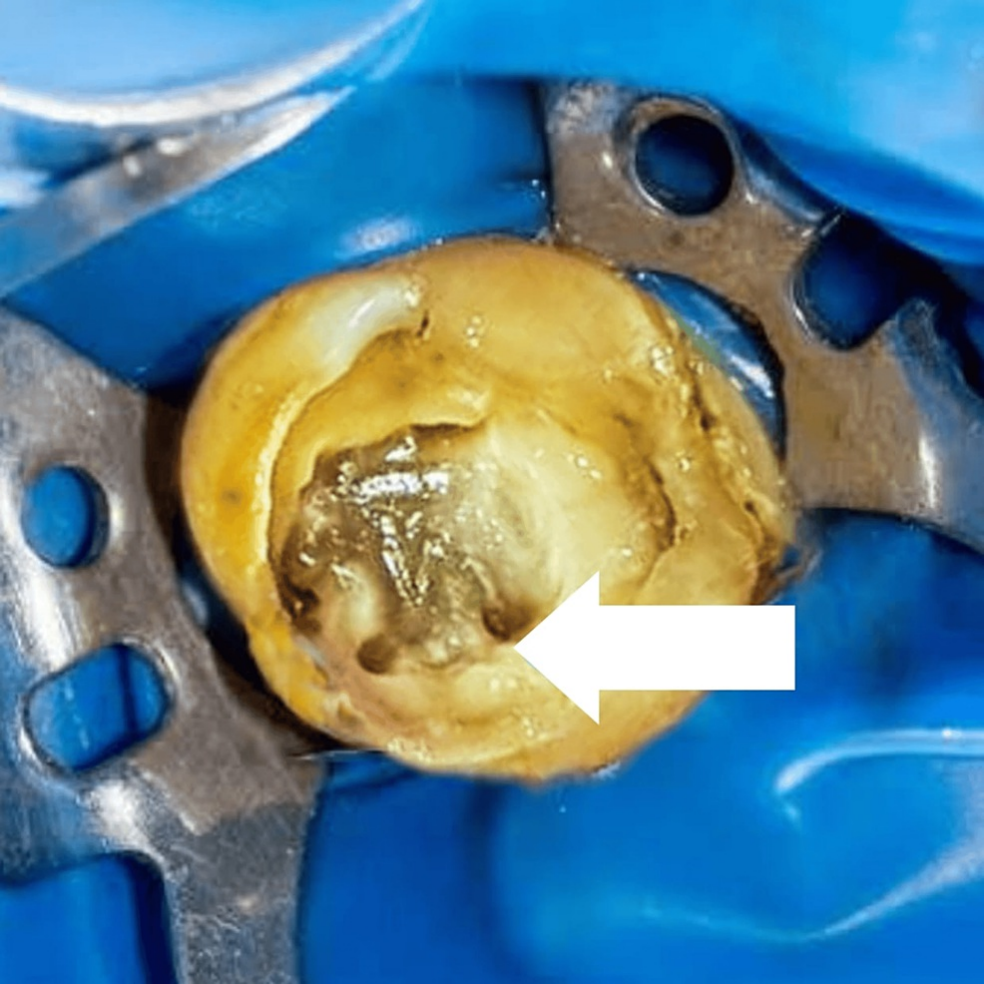
Access opening with the upper right second molar showing the distopalatal canal

Xylocaine 2% with 1:80,000 adrenaline (Xylocaine 2% injection, Zydus Cadila, India) was administered to the patient. Isolation was done with the help of a rubber dam. The access opening was done with Round BR-45 (Mani, Japan) and Safe End bur EX-24 (Mani, Japan). When the pulp tissue was removed from the pulp chamber, four openings were discovered: mesiopalatal, mesiobuccal, and distobuccal openings in typical places, and a bleeding point was seen on the distal side of the access cavity. The mesial and the distal canals in the mesial and the distal roots are joined with the help of the groove, which confirms the presence of individual palatal canals on the palatal side, corresponding to Vertucci's type I classification. To facilitate easier access to the extra canal, the conventional triangular access was modified to a trapezoidal shape (Figure 2).

Working length was determined using the Root ZX mini apex locator (JW Morita, Japan) and the readings were mesiopalatal, 21 mm; distobuccal, 20.5 mm; mesiobuccal, 18 mm; and distopalatal, 18.5 mm.

**Figure 3:**
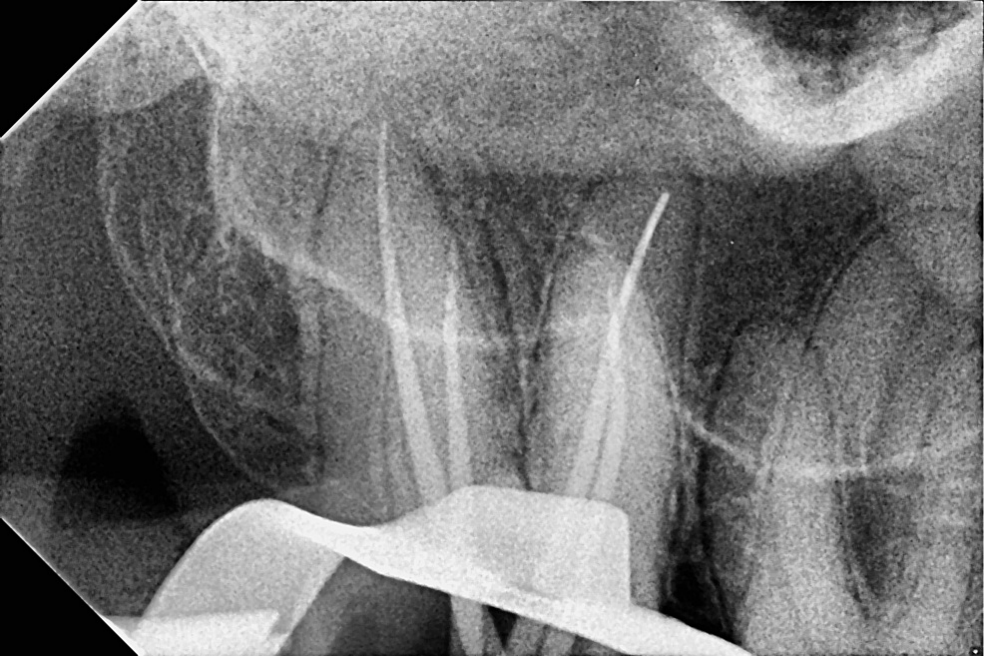
Mastercone fit check with the upper right second molar using radiovisiography (RVG)

Biomechanical preparation of the canals was done using rotary Nickel-Titanium (Woodpecker 2019, China) files up to 20-6% in the mesiopalatal canal, 20-6% in the mesiobuccal and distobuccal, and 20-6% in the distopalatal canal. The canal was irrigated using 3% NaOCl and 0.9% saline alternatively. Calcium hydroxide (RC Cal, Prime, India) temporary closed dressing (Neotemp, Neoendo, India) was given, and the patient was further recalled after seven days. On the second appointment, the patient was completely devoid of symptoms. The temporary dressing was removed, and all the canals were sonically activated with 17% EDTA to facilitate calcium hydroxide removal. All the canals were then irrigated using 3% NaOCl and 0.9% saline alternatively. Gutta-percha master cones were selected (Figure 3).

**Figure 4:**
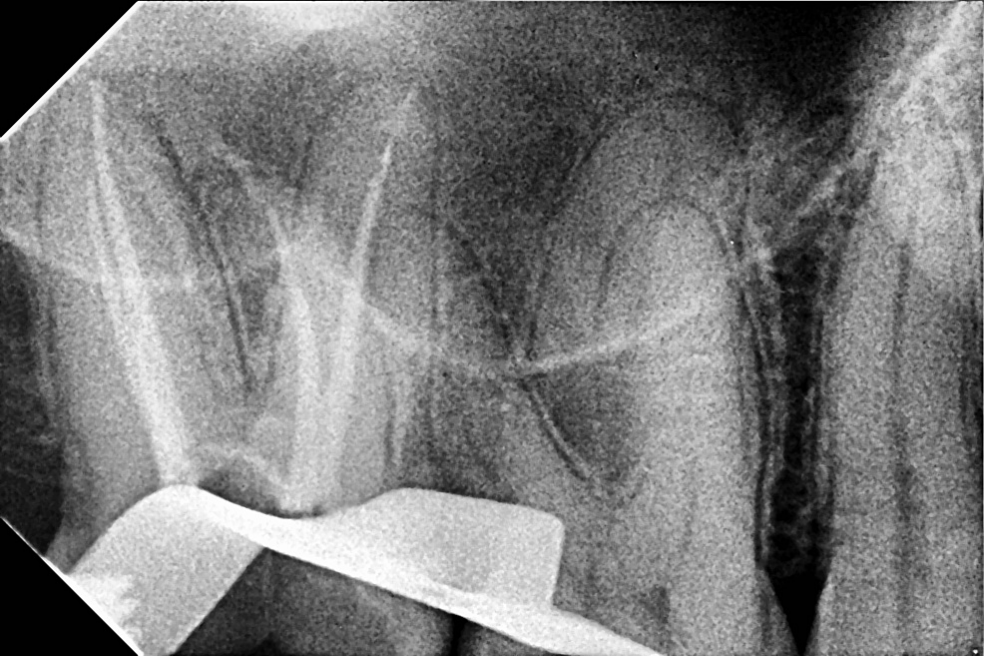
Obturation radiograph of the upper right second molar

Obturation was carried out with master cones and epoxy resin-based sealer (Diaproseal, Diadent, Canada) (Figure 4).

**Figure 5:**
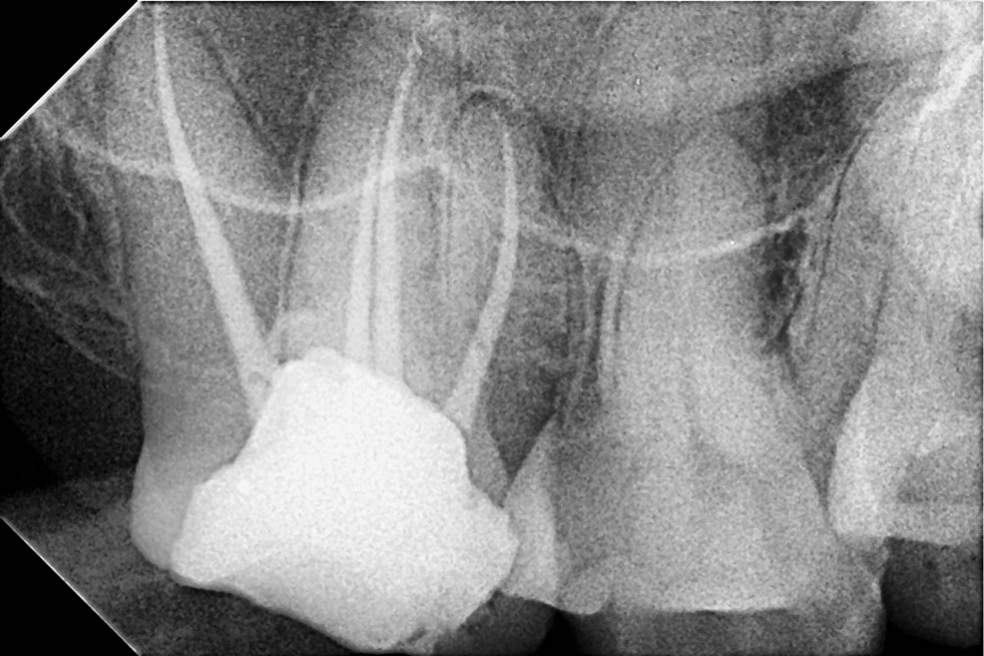
Post-endodontic restoration of the upper right second molar

Post-endodontic composite restoration (Spectrum, Dentsply, USA) was done (Figure 5).

## Discussion

In the case of maxillary second molars with two palatal canals, these canals can often exhibit significant curvature. Identifying the curvature of these canals is vital, as highly curved canals require careful navigation to avoid procedural errors such as ledging or perforation. Advanced diagnostic tools such as CBCT can be extremely helpful in assessing the root canal anatomy and curvature before treatment. In this case report, two palatal canals have distinct orifices, and the access cavity was modified to create separate openings for each canal. The access cavity was extended more towards the palatal aspect. This allows for better visualization and instrumentation of each canal independently.

In the literature, four-root maxillary second molars are not frequently observed. Alavi et al. studied the morphology of the root and its canal of 268 molars in a population of the Thai region but found no molars having four roots [7]. Peikoff et al. found a second palatal root in 1.4% of upper molars [8]. Hartwell et al.'s in vivo study showed that 9.6% of 176 upper second molars had four canals [9].

**Figure 6:**
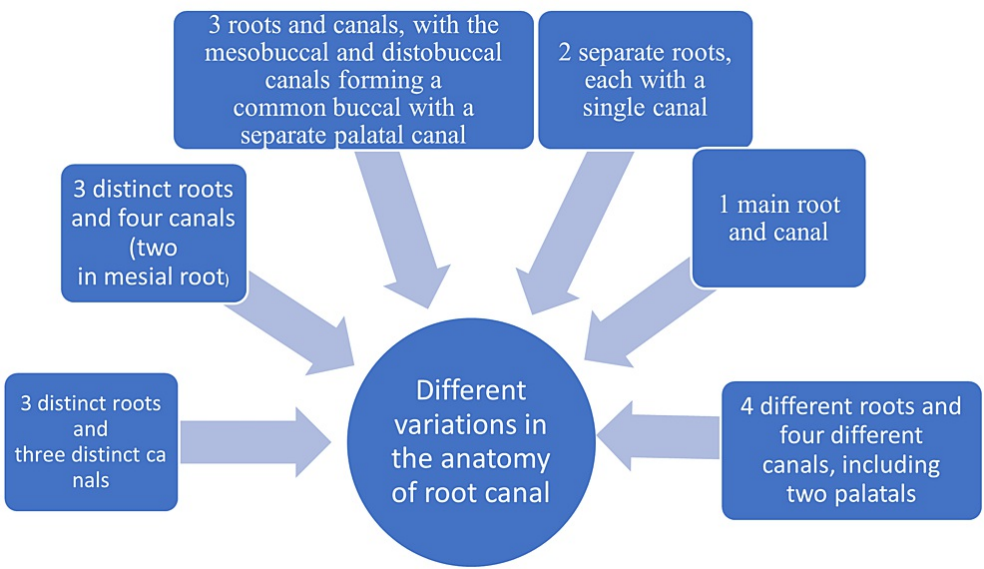
Anatomical variations of root canals Source: Ref. [8]

Peikoff et al. [8] performed a retrospective study of 520 upper second molars completed with root canal treatment and classified them into six categories (Figure 6).

Besides the findings of Peikoff et al., there are also some uncommon variants of maxillary second molar. Yang et al. found that the upper second molars had a C-shaped root in 4.5% and a C-shaped root opening with a C-shaped root in 4.9% of the Chinese population [10]. This report defines four separate canals: a mesiobuccal root with the mesiobuccal canal, a palatal root with mesiopalatal and distopalatal separate canals, and a distobuccal root with a distobuccal canal.

After thoroughly cleaning and shaping the canals, obturation should be performed with materials that can effectively seal the complex canal anatomy. Thermoplasticized gutta-percha techniques, such as continuous wave compaction or the use of bioceramic sealers, are highly effective in achieving a three-dimensional seal. Finally, the success of the endodontic treatment heavily relies on the quality of the post-endodontic restoration. A well-sealed coronal restoration is essential to prevent microleakage and reinfection.

The abnormality in the upper second molar's anatomy makes it challenging to diagnose due to its posterior location. Exposure to several X-rays taken from different angles can help eliminate overlap and allow the doctor to detect this rare abnormality. Accessing the root canal is the first step in preparing a root canal. Access cavities designed and prepared correctly can prevent many potential issues during canal preparation and filling. Magnification devices such as dental microscopes and loupes allow for better visualization and location of anatomical abnormalities. In the present case, 3.5x dental loupes are used for magnification and illumination purposes. The magnification provides a significantly enlarged and clearer view of the treatment area. This enhanced visualization allows clinicians to identify subtle anatomical features and distinguish between canal orifices more effectively.

## Conclusions

The presence of two palatal canals in maxillary second molars, often referred to as "palatal radiculous" or "C-shaped canals," is a relatively rare anatomical variation. When encountered, it can pose challenges during endodontic treatment due to the complexity of the root canal system. However, with advancements in dental imaging techniques and instrumentation, clinicians are better equipped to identify and manage such cases. Clinically, careful examination and diagnostic imaging, such as CBCT, are essential for detecting the presence of additional canals in maxillary second molars. Previous literature suggests that, with proper detection and treatment, the prognosis for teeth with an additional palatal canal is favorable. Accurate identification and meticulous management are crucial to ensuring successful endodontic outcomes and preserving tooth volume.
